# Elucidating interfacial-function relationships of lactoferrin-cellulose nanofibril complexes via multidimensional spectroscopy and QCM-D: interfacial anchoring and bulk stabilization mechanisms in robust Pickering emulsions

**DOI:** 10.1016/j.fochx.2026.104455

**Published:** 2026-09-19

**Authors:** Runru Xiang, Jiwei Ding, Xiaohui Wan, Yufei Yang, Wentao Zheng, Mo Li, Tao Ma, Yongfeng Liu

**Affiliations:** a College of Food Engineering and Nutritional Science, Shaanxi Normal University, Xi'an 710119, Shaanxi, China; b College of Food Science and Nutritional Engineering, China Agricultural University, Beijing 100083, China

**Keywords:** Lactoferrin, Cellulose nanofibril, Quartz crystal microbalance, Interaction, Interfacial behavior

## Abstract

Pickering emulsions stabilized by natural biopolymers have garnered significant interest due to their safety and functionality. In this study, lactoferrin (LF) and cellulose nanofibril (CNF) were complexed via electrostatic interactions under varying pH conditions. Multidimensional spectroscopic and interfacial tension analyses revealed that LF dominates the interfacial behavior of the complexes. QCM-D demonstrated that LF-CNF complexes formed a denser interfacial layer (apparent adsorbed mass ∼ 287.0 ng/cm^2^), approximately 1.8 times higher than LF alone (∼159.4 ng/cm^2^) and 12 times higher than CNF alone (∼22.5 ng/cm^2^). When used as Pickering emulsion stabilizers, all emulsions exhibited shear-thinning behavior. The 1.0 wt% LF-CNF complexes provided the finest droplet size, highest uniformity, and longest storage stability (up to 28 days). This study clarifies the interaction mechanisms and interfacial behavior of protein-polysaccharide complexes, offering theoretical guidance for designing high-performance food-grade emulsifiers with natural origin.

## Highlights

• LF–CNF complexes formed via electrostatics are optimal at pH 6, 1:1 ratio.
• LF-CNF complexes form an dense interfacial layer (287.04 ng/cm^2^).
• LF–CNF emulsions showed shear-thinning, improved viscoelasticity, 28 d stability.
• wt% LF–CNF gave uniform droplets, stable at pH 4–10 and ≤ 100 mM NaCl.

## 1 Introduction

In contrast to traditional emulsions, Pickering emulsions have attracted considerable attention in areas like food, pharmaceuticals, cosmetics, and materials science due to their superior physical stability and good biocompatibility imparted by solid particle interfacial stabilization (Guo et al., 2021; Peito et al., 2022). Unlike conventional surfactant-stabilized emulsions, Pickering emulsions achieve stability through the adsorption of solid particles at the interface between oil and water, where stability is primarily governed by steric hindrance effects (Ma et al., 2021). With the growing emphasis on “clean-label” products and sustainable development, natural biopolymer-based particles have attracted significant research attention in this field (Rayees et al., 2024).

Proteins are considered ideal natural emulsifying agents because of their abundant availability, significant nutritional value, and excellent biocompatibility (Shen et al., 2026). Milk-derived globular proteins, including whey protein, β-lactoglobulin, α-lactalbumin, and lactoferrin, are commonly used to stabilize oil-in-water (O/W) emulsions (Tokle & McClements, 2011; Yuliarti et al., 2019). However, individual proteins still exhibit several limitations in practical applications, including insufficient interfacial stabilization capacity, poor wettability, and susceptibility to conformational changes under extreme pH, high temperature, or high ionic strength conditions, which may result in emulsion instability (Zhao et al., 2022). To overcome these limitations, protein–polysaccharide complexes have attracted increasing attention. These complexes possess good biocompatibility and biodegradability, and can form more robust interfacial adsorption layers through synergistic interactions, thereby markedly improving the dynamic stability of emulsions (Zhang et al., 2025). For instance, although whey protein isolate is an amphiphilic biopolymer, its use alone in stabilizing Pickering emulsions often causes in droplet clustering and diminished stability (Bai et al., 2026). However, its emulsifying performance can be significantly enhanced through the formation of complexes with polysaccharides like chitosan, Tremella polysaccharides, or cellulose nanocrystals (Tian et al., 2023; Yildiz & Altintas, 2026; Yu et al., 2024). Therefore, the development of such complexes as stabilizers for Pickering emulsions holds significant research value and promising application potential.

Lactoferrin is a glycoprotein characterized by its strong iron-binding capacity, with a molecular weight of approximately 80 kDa. Its elevated isoelectric point (pI >8) results in a positive charge at neutral pH, making it prone to binding with anionic polysaccharides through electrostatic interactions. Lactoferrin provides not only outstanding nutritional benefits but also displays various biological functions, including antioxidant, antibacterial, and immunomodulatory effects, indicating considerable promise in the areas of functional foods and drugs (Cao et al., 2023). Meanwhile, nanocellulose, as a type of renewable green nanomaterial, has garnered significant attention in Pickering emulsions (Zhao et al., 2024). Compared to cellulose nanocrystals (CNC), although cellulose nanofibril (CNF) are capable of stabilizing O/W emulsions, their larger size and strong hydrophilicity result in poor performance in stabilizing Pickering emulsions (Ji & Wang, 2023; Ma et al., 2022). Although LF and CNF each demonstrate potential as natural emulsifying components, a single component often struggles to simultaneously achieve strong interfacial adsorption and a robust spatial barrier. By constructing LF-CNF complexes, it is expected that interfacial behavior and spatial stabilization can be synergistically regulated at the molecular level. Unlike previously reported CNC-protein complexes, the LF-CNF complexes represent a distinct protein-cellulose assembly strategy. The flexible fibrillar structure of CNF may enable LF-CNF complexes to provide not only interfacial anchoring but also continuous-phase network reinforcement, thereby contributing to emulsion stability through dual mechanisms. However, there is still an insufficient detailed explanation concerning of in-depth elucidation regarding the interaction mechanisms between LF and CNF, the adsorption kinetics of these complexes at the oil-water interface, and the systematic regulatory principles governing their effects on the stability of Pickering emulsions.

Therefore, this study aims to synthesize LF-CNF complexes and systematically investigate their formation mechanisms, interfacial properties, and emulsifying performance. Specifically, we will employ techniques such as one-dimensional and two-dimensional Fourier transform infrared spectroscopy to elucidate the interaction mechanisms between LF and CNF. Additionally, we will apply quartz crystal microbalance (QCM-D) along with interfacial tension measurements to uncover the adsorption characteristics of the complexes at the oil-water interface. Ultimately, we will create O/W Pickering emulsions using the LF-CNF complexes as a stabilizer, systematically investigate their microstructure, rheological properties, and long-term physical stability, as well as assessing the influence of environmental variables. This study offers innovative material options and a theoretical foundation for creating high-performance, environmentally friendly “clean label” emulsion products.

## 2 Materials and methods

### 2.1 Materials

Cellulose nanofibril (CNF) was acquired from Guilin Qihong Technology Co., Ltd. (Guangxi, China), while lactoferrin (LF, 95% purity) was sourced from Shanghai Yuanye Biotechnology Co., Ltd. (Shanghai, China). The corn oil was obtained locally in Xi'an, China. The fluorescent dyes, including Nile Red, Nile Blue, and Calcofluor White, were purchased from Xi'an Jingbo Biotechnology Co., Ltd. (Xi'an, China).

### 2.2 Preparation of LF-CNF complexes

LF powder was mixed with water and agitated for two hours to achieve homogeneously dispersed LF solutions at concentrations of 0.2, 0.4, 0.6, 0.8, and 1.0 wt%. CNF was stirred in water for 30 min, then sonicated at 200 W for 5 min (3 s on, 2 s off) using an ultrasonic cell disruptor to obtain well-dispersed CNF dispersions at 0.2, 0.4, 0.6, 0.8, and 1.0 wt%. Subsequently, the pH of the LF and CNF solutions were modified to fall within the range of 2–9. Equal volumes of both dispersions at matching pH levels were then mixed and agitated for 2 h to form LF-CNF complexes across the varying pH levels.

### 2.3 Characterizations of LF-CNF complexes

#### 2.3.1 Field-emission transmission electron microscope (FETEM)

The sample microstructure was characterized by FETEM (Tecnai G2 F20, FEI, USA). Prior to analysis, CNF and LF were each diluted to 0.5 wt%. They were then individually placed onto copper grids and left to sit for 2 to 3 min. Following this, the samples were stained with 2.0 wt% phosphotungstic acid for approximately 10 min. After staining, the samples were air-dried completely prior to observation. ImageJ was employed to process the obtained micrographs and quantify the distributions of particle diameter and length. The TEM images (Fig. S1) revealed that LF exhibited a typical spherical morphology, whereas CNF displayed a slender fibrillar structure. Measurements performed using ImageJ indicated that the the diameter of LF was 32.93 ± 5.8 nm, while the CNF exhibited a diameter of 8.72 ± 2.9 nm and a length of 224.28 ± 75.7 nm.

#### 2.3.2 Zeta potential

The zeta potentials of LF, CNF, and their complexes (LF-CNF) were determined at various pH values using a NanoBrook 90 Plus PALS analyzer (Brookhaven Instruments, USA).

#### 2.3.3 Contact angle

The contact angle goniometer (KRUSS DSA100, KRUSS, Germany) was employed to determine the contact angles of CNF, LF, and LF-CNF complexes prepared at varying mass ratios. First, the freeze-dried CNF, LF, and LF-CNF complexes were made into thin films using a tablet press. Then, a 1.5-mm needle was used to drop a water droplet onto the thin film. Images were recorded by a high-speed camera.

#### 2.3.4 Intrinsic fluorescence characterization

Fluorescence spectra of LF and LF-CNF complexes were recorded with a spectrofluorometer (RF-6000, Shimadzu, Japan). The excitation wavelength was set at 280 nm, while emission spectra were gathered within the range of 300 to 500 nm.

#### 2.3.5 Fourier transform infrared spectroscopy test

Structural characteristics of CNF, LF, and LF-CNF systems at pH 6.0 were examined using a FTIR spectroscopy (Invenio S, Bruker, UK) equipped with a TR module was employed to analyze the structures of CNF, LF, and LF-CNF mixtures at various concentrations at pH 6.0. The spectral data were recorded across the wavenumber interval spanning 500 to 4000 cm^−1^.

The one-dimensional FTIR spectra of all samples were subjected to automatic baseline correction and deconvolution, and the processed data were subsequently used to generate two-dimensional correlation FTIR (2D-COS-FTIR) spectra.

#### 2.3.6 Interfacial tension measurement

The interfacial tension was measured by the pendant drop method using an optical contact angle measuring instrument (KRUSS - DSA100, KRUSS, Germany). A pendant drop was created using a needle that had a diameter of 1.80 mm. After the droplet reached an equilibrium state, it was left to stand for 10 min before the image was collected. The value of interfacial tension was calculated by fitting the shape of the droplet according to the Young-Laplace equation. All tests were carried out at pH 6.0, and the specific sample systems were as follows:

(1) LF and CNF dispersions at concentrations of 0.2, 0.4, 0.6, 0.8, and 1.0 wt%;

(2) With the CNF concentration fixed at 1.0 wt%, the LF was incrementally raised from 0.2 wt% to 1.0 wt%;

(3) Keeping the CNF level constant at 1.0 wt%, CNF was gradually added with its concentration ranging from 0.2 to 1.0 wt%.

#### 2.3.7 QCM-D

A QCM-D was utilized to examine the interactions between LF and CNF, along with the adsorption behavior of LF, CNF, and the LF-CNF complexes on the surface of corn oil. Prior to each use, the gold sensor chips (QSX 301) were initially soaked in a 2.0 wt% SDS solution, left at room temperature for 35 min, then rinsed thoroughly with an abundance of deionized water, and finally dried with pure, oil-free nitrogen for later use.

(1) Interaction between LF and CNF: A prepared 1.0 wt% CNF solution was spin-coated on the surface of the gold sensor chips (4000 rpm, 40 s), followed by drying to promote the formation of a uniform and stable CNF layer on the surface. Then, a prepared 1.0 wt% LF solution was introduced into the system;

(2) Adsorption behavior of LF, CNF, and LF-CNF complexes on the corn oil surface: Prior to testing, corn oil diluted with chloroform (0.5%, *w*/*v*) was spin-coated onto the chip surface using a spin coater (3000 rpm, 40 s), followed by drying to promote the formation of a uniform and stable oil film on the chip surface. Subsequently, prepared 1.0 wt% solutions of LF, CNF, and the LF-CNF complexes were introduced into the system.

QCM-D analysis was performed according to the method of Hu et al.(Hu et al., 2022). In brief, the frequency shifts (*Δf*) at multiple overtones were monitored during the adsorption process and processed using Dfind software. The apparent adsorbed mass was estimated according to the Sauerbrey equation:

(1) Δm=−C/n·Δf

where *C* represents the mass sensitivity constant (17.7 ng cm^−2^ Hz^−1^), and n represents the harmonic number. The fifth overtone was selected for quantitative comparison because it provided a stable frequency response with relatively reduced instrumental noise. The calculated values were used for comparative evaluation of adsorption differences among samples rather than absolute mass quantification.

#### 2.3.8 Interfacial thickness

The interfacial layer thickness was estimated following the approach of Zhao et al. (Zhao et al., 2025). Briefly, 900 μL of complexes solutions at different concentrations was mixed with 100 μL of 0.1 wt% polystyrene latex microsphere suspension and stand for half hour to reach equilibrium. The particle size of the latex microspheres was then measured before and after the formation of the interfacial layer. The interfacial thickness were calculated using the following equation:

(2) $$\begin{array}{c} \text{Interfacial layer thickness}=\left(D_{1}-D_{0}\right)/2 \end{array}$$

Where D_0_ and D_1_ represent the particle diameters of the polystyrene latex microspheres before and after adsorption, respectively.

### 2.4 Preparation of emulsion

LF-CNF complexes at concentrations of 0.2, 0.4, 0.6, 0.8, and 1.0 wt% were used as the aqueous phase, while corn oil served as the oil phase. Emulsions were prepared using ultrasonication with an oil-to-water ratio of 1:9. Each sample was sonicated at 200 W for 5 min (3 s on, 2 s off, in cycles). The complete procedure was conducted in an ice bath to avoid overheating of the probe in localized areas. The emulsions that were prepared were stored at 4 °C for later experiments.

### 2.5 Characterizations of emulsions

#### 2.5.1 Droplet size and physical stability of emulsion

The prepared emulsions were transferred into glass containers and stored at 4 °C for a period of 28 days. Throughout this storage period, its visual characteristics were regularly monitored and documented through photographs. The droplet size of the emulsion on day 0 and day 28 was measured using a Laser particle sizer (MS3000, Malvern, UK). The physical stability of the emulsion was evaluated using an emulsion stability analyzer (Turbiscan tower, Formulaction, France). An appropriate amount of sample was placed in the analysis bottle, and the emulsion stability was measured by scanning once every 2 min.

#### 2.5.2 Rheological behaviors

The rheological properties of the emulsions were analyzed utilizing a rheometer (MCR 302e, Anton Paar, Austria). Apparent viscosity was assessed with a 40 mm plate across shear rates from 10^−2^ to 10^2^ s^−1^. To investigate the viscoelastic properties of the emulsion, the loss modulus (G″) and storage modulus (G′) were evaluated using frequency sweep testing (sweep frequency range: 10^−2^ to 10^2^ Hz, strain level: 0.01%).

#### 2.5.3 Confocal laser scanning microscope (CLSM)

CLSM was employed to examine and visualize the microstructural features of the emulsions. 1 mL of the emulsion sample was mixed with 10 μL of the oil phase staining solution Nile Red (1 mg/mL), 10 μL of the protein staining solution Nile Blue (1 mg/mL), and 10 μL of the polysaccharide staining solution Calcofluor Fluorescent Brightener (1 mg/mL). All procedures were conducted under light-protected conditions, and the mixture was allowed to stand for 20 min prior to measurement. Subsequently, 2 μL of the stained emulsion sample was dropped onto a microscope slide, and a cover slip was carefully placed on top to avoid air bubble formation. Diode lasers with excitation wavelengths of 405 nm, 488 nm, and 637 nm were selected, and observations were made under a 60 fold oil immersion objective. The captured images were analyzed with FLUOVIEW Viewer software to obtain merged complexes images from three different channels.

#### 2.5.4 Cryo-SEM

The cryo-scanning electron microscope (Hitachi Regulus 8100, Japan) was employed to examine the microstructure of the samples. After undergoing ultra low temperature freezing, fracturing, and gold sputtering treatments, the samples were positioned on the cold stage inside the electron microscope through a cryo transfer system for observation.

#### 2.5.5 Influence of pH and ionic concentration on emulsion

To systematically assess how environmental conditions influence emulsion stability, Pickering emulsions stabilized with 1.0 wt% LF-CNF complexes were used to examine the influence of pH and ionic concentration. The pH levels of the emulsion were modified to 2.0, 4.0, 6.0, 8.0, and 10.0 through the addition of HCl or NaOH solutions. For the ionic strength treatments, NaCl was added to achieve final concentrations of 20, 40, 60, 80, and 100 mM. Under each condition, the emulsions were assessed in terms of their visual appearance and droplet size distribution.

### 2.6 Statistical analysis

Experimental data were collected in triplicate, and the mean value along with the standard deviation was determined. Statistical analysis of the data was performed using SPSS Statistics 26 software. Letters that differ denote statistically significant differences among the variables (*p* < 0.05).

## 3 Results and discussion

### 3.1 Bulk analysis of LF, CNF, and LF-CNF complexes

Zeta potential was used to assess the electrostatic interactions between proteins and polysaccharides. The attraction between molecules with different charges determines the likelihood and strength of intermolecular interactions (Tian et al., 2024). A complex system is generally acknowledged to maintain a degree of stability when its absolute zeta potential is above 20.0 mV (Ma et al., 2021). Based on this, the zeta potentials of LF, CNF, and LF-CNF complexes were determined under various pH conditions. Fig. 1A shows the variation of the zeta potential of LF with pH. Specifically, LF exhibits a positive zeta potential at pH values under 8, while it becomes negative when the pH exceeds 8. This behavior is due to lactoferrin being a basic protein characterized by a notably high isoelectric point (pI >8.0) (Ma et al., 2021; Tokle & McClements, 2011). Meanwhile, as illustrated in Fig. 1B, CNF maintains a negative zeta potential throughout the pH range of 2.0–9.0. This may be because the carboxyl groups (-COOH) on the CNF surface dissociate at different pH levels, resulting in a change in potential (Alves et al., 2020). Subsequently, the zeta potential of LF-CNF complexes was evaluated over this pH range. As shown in Fig. 1C, the complexes were positively charged at pH 2.0, whereas they carried negative charges at pH values above 2.0. As the pH rises, the absolute zeta potential value steadily ascended, reaching a maximum of 33.10 mV at pH 6.0. The variation trend of the complexes were generally consistent with that of CNF. This is likely because CNF is attached to the surface of LF, causing the complexes to exhibit charge properties nearly identical to those of CNF (Xu et al., 2019).

**Fig. 1:**
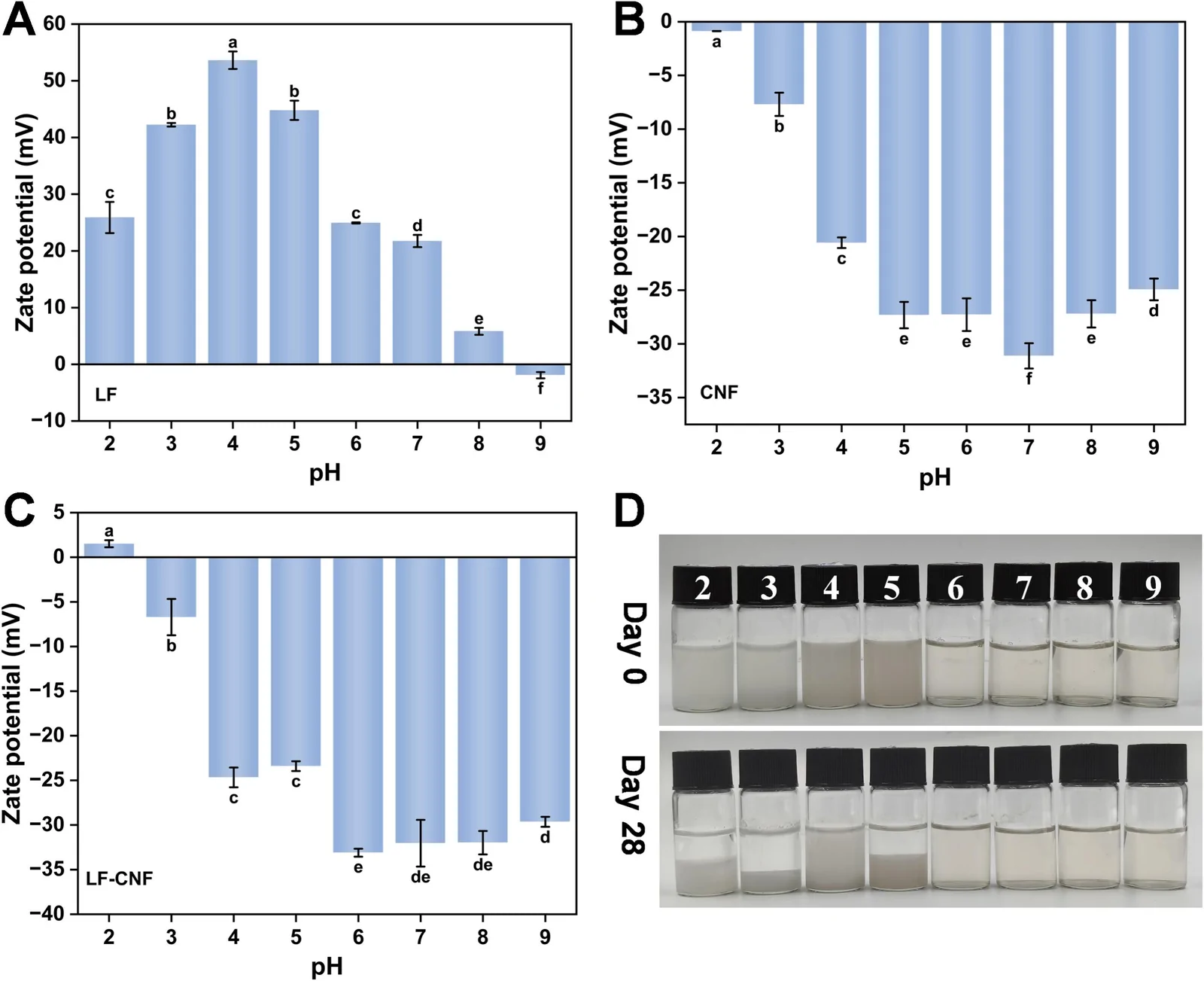
(A–C) Zeta-potentials of LF, CNF, and LF-CNF complexes; (D) Visual appearance.

To assess the macroscopic stability of the complexes across various pH levels, their visual appearance was observed at day 0 and after 28 days of storage (Fig. 1D). The results indicated that within the pH range of 2.0–5.0, the complexes exhibited varying degrees of delamination, suggesting poor physical stability under these conditions. This macroscopic phenomenon corroborates the previously measured zeta potential results. Considering both the Zeta potential and macroscopic stability, it can be concluded that the complexes prepared at pH 6.0 has the highest absolute Zeta potential and a uniform appearance within 28 days, demonstrating the best stability. Therefore, considering the favorable electrostatic compatibility between LF and CNF as well as the good macroscopic stability of the resulting complexes, pH 6.0 was selected as the optimal condition for LF-CNF complex formation. Subsequently, the interactions and structural characteristics of the LF-CNF complexes formed under this condition were further investigated.

To further evaluate the effect of the LF-CNF ratio on the complexes, we measured the contact angles of LF, CNF, and their complexes prepared at mass ratios of 10:1, 5:1, 2:1, and 1:1 (CNF:LF) at pH 6.0. As shown in Fig. 2A, the measured contact angles were 41.5° for pure LF and 45.2° for CNF, respectively, both exhibiting hydrophilic properties. Nonetheless, the contact angles observed for all the LF-CNF complexes surpassed those of the individual components, and there was an increase in the contact angle corresponding to a higher proportion of LF within the complexes. When the LF:CNF mass ratio was 1:1, the contact angle peaked at 72.3°, demonstrating that the addition of LF significantly influences the surface wettability of the complexes. In addition, we evaluated the stability of complexes with different mass ratios (Fig. 2C). The results indicated that the complex with a 1:1 mass ratio had the smallest stability index, indicating the strongest physical stability. Considering both wettability and stability, the LF-CNF complexes at a 1:1 mass ratio exhibited the optimal overall performance. Therefore, all subsequent experiments in this study were conducted using this ratio (LF:CNF = 1:1).

**Fig. 2:**
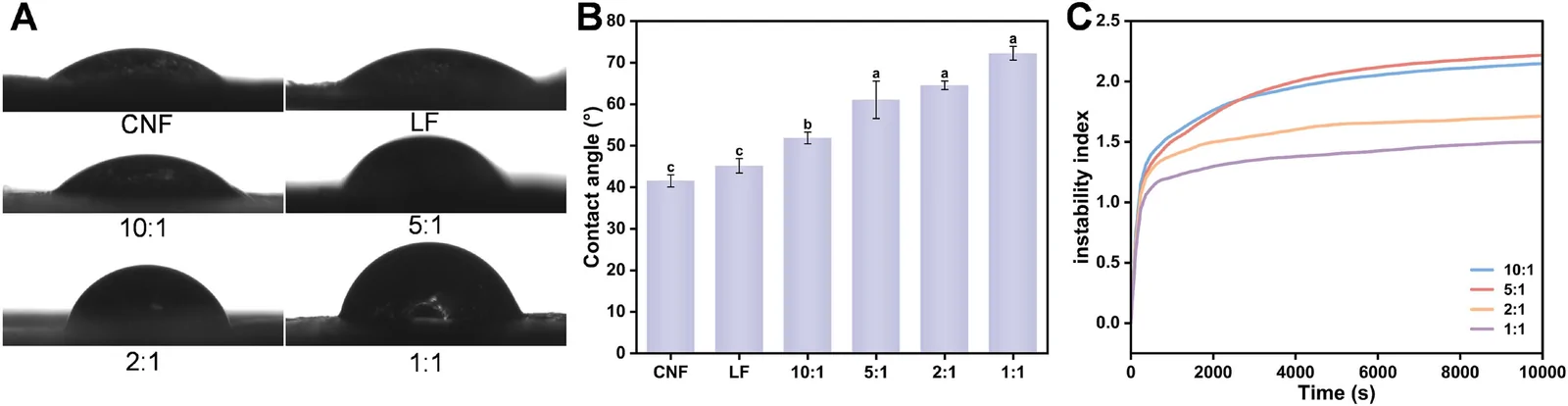
(A and B) Contact angles of LF-CNF complexes with different LF:CNF mass ratio of 10:1, 5:1, 2:1, and 1:1; (C)The physical stability.

### 3.2 Interactions between LF and CNF

#### 3.2.1 Intrinsic fluorescence

Protein intrinsic fluorescence mainly arises from the presence of aromatic amino acid residues within their molecular structure, including tryptophan, tyrosine, and phenylalanine. Among these, the fluorescence signal of LF is predominantly attributed to its tryptophan residues (Lin et al., 2026). To this end, a comparative analysis of the intrinsic fluorescence spectra of LF and LF-CNF complexes was conducted to clarify their influence on the tertiary structure of LF. As shown in Fig. 3A, the maximum emission wavelength of both LF and the LF-CNF complexes remained at 328 nm, indicating that no significant alteration occurred in the tertiary structure of LF (Xu et al., 2019). However, compared with LF alone, a marked increase in fluorescence intensity was observed for all complexes. This enhancement may be attributed to the electrostatic attraction between positively charged LF and negatively charged CNF, which could induce local conformational rearrangements of LF and alter the microenvironment of tryptophan residues, thereby increasing fluorescence intensity (Ding et al., 2025). Nevertheless, considering the nanofibrous nature of CNF, the potential contribution of light scattering effects to fluorescence intensity changes cannot be completely ruled out. Furthermore, as the concentration of the complexes increased, the fluorescence intensity gradually decreased, which is likely due to fluorescence self-quenching caused by protein aggregation resulting from LF-CNF interactions (Chen et al., 2017).

**Fig. 3:**
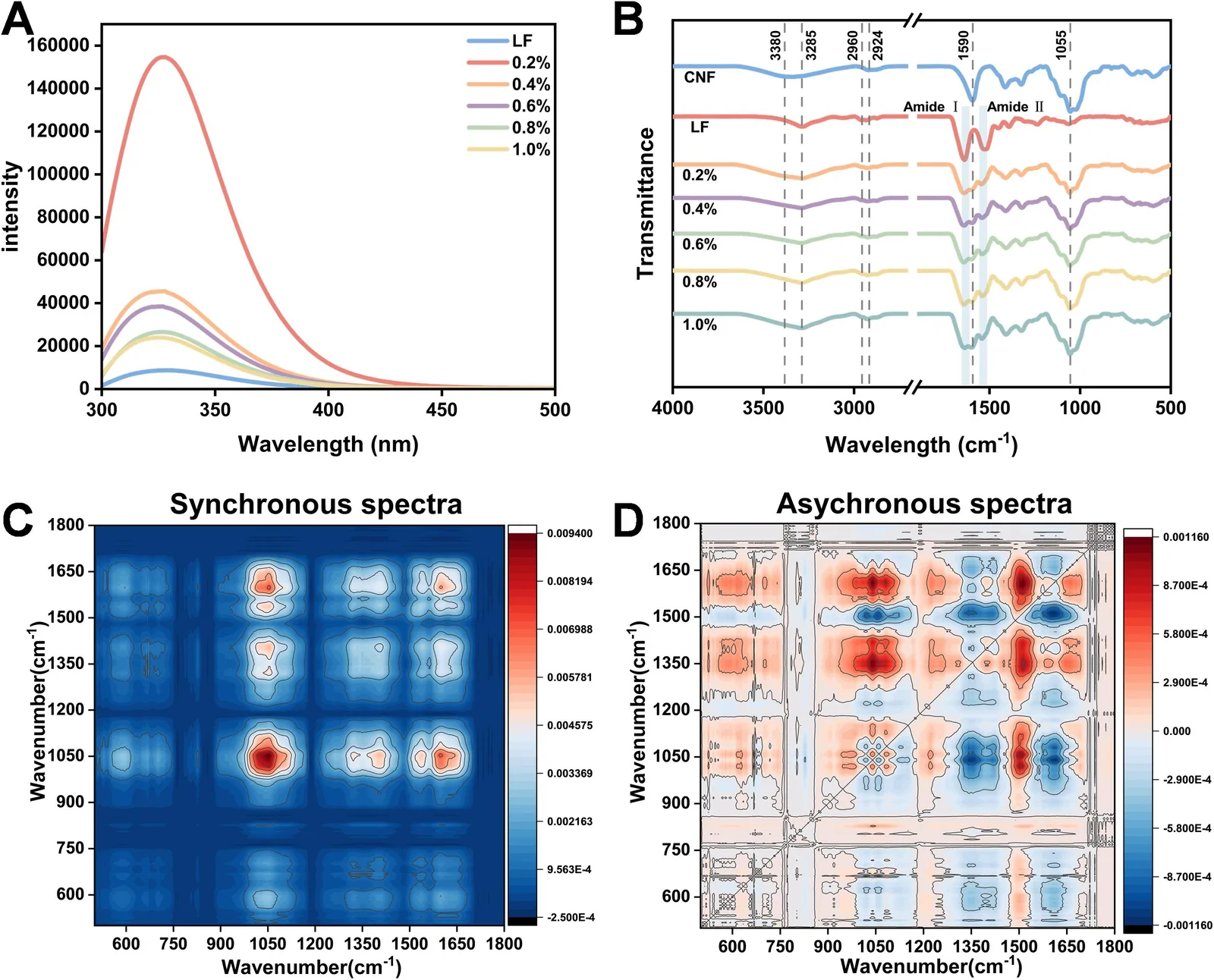
(A) Intrinsic fluorescence spectra of LF and LF-CNF complexes; (B) FTIR spectra of CNF, LF and LF-CNF complexes; (C and D) 2D-COS-FTIR Analysis of the Complexes.

#### 3.2.2 FTIR

As shown in Fig. 3B, FTIR analysis was conducted to examine structural variations in LF, CNF, and their LF-CNF complexes. Both LF and CNF exhibit common absorption features, including a wide band at 3600–3100 cm^−1^ (O—H bond stretching vibration) and a peak at 3000–2800 cm^−1^ (-CH_2_ stretching vibration). LF exhibits typical protein characteristic peaks at 1700–1600 cm^−1^ (amide I band, primarily corresponding to C=O stretching vibrations) and 1580–1480 cm^−1^ (amide II band, primarily corresponding to N—H bending vibrations) (Meng et al., 2024). CNF exhibits a distinct absorption peak at approximately 1590 cm^−1^, which is due to the asymmetric stretching of C=O in carboxylate groups. Additionally, there is a peak close to 1055 cm^−1^ that relates to the stretching vibration of C-O-C, a characteristic peak associated with cellulose (Jiang & Hsieh, 2016). For the LF-CNF complexes, the O—H stretching vibration band exhibited noticeable broadening, indicating changes in the hydroxyl group environment and suggesting the possible involvement of hydrogen bonding interactions during complex formation (Zhang et al., 2022). The amide I band showed a slight shift and broadening after interaction with CNF, suggesting alterations in the local environment of protein amide groups and possible conformational rearrangements of LF (Li et al., 2025). Furthermore, the characteristic peaks of CNF at 1055 cm^−1^ and 1590 cm^−1^ were retained in the complexes, indicating the successful incorporation of CNF into the LF-CNF complexes. Notably, no new characteristic absorption peaks were detected, suggesting that no obvious new chemical bonds were generated within the detection limit of FTIR and that the complex formation was mainly governed by non-covalent interactions, including hydrogen bonding and electrostatic interactions.

To gain deeper insights into how concentration influences the structure of LF-CNF complexes, FTIR data were analyzed using generalized two-dimensional correlation spectroscopy. Compared to one-dimensional spectroscopy, 2D-COS-FTIR improves spectral resolution and reveals the response sequence and correlations of functional groups through synchronous and asynchronous spectra, providing more detailed structural information (Li et al., 2015). Based on the one-dimensional FTIR spectra (Fig. 3B), obvious spectral changes among LF, CNF, and LF-CNF complexes were mainly observed in the region of 500–1800 cm^−1^. This region includes characteristic vibrations of protein amide groups and cellulose-related functional groups, which are closely associated with intermolecular interactions during complex formation. Therefore, the 2D-COS-FTIR analysis was performed within this region to provide further insight into the sequence of spectral variations and the interaction mechanism between LF and CNF. As shown in Fig. 3C and D, the spectra are presented as contour plots, where red indicates positive correlations and blue denotes negative correlations. In the synchronous spectra (Fig. 3C), the diagonal features (autopeaks) indicate how responsive the absorption intensity at specific wavenumbers is to variations in external conditions, a higher intensity indicates a more pronounced response of the functional group. The off-diagonal peaks (cross peaks) indicate whether the vibrational modes at two wavenumbers change in synchrony (Peng et al., 2025). The asynchronous spectra (Fig. 3D) contain only cross peaks and are used to determine the response sequence of functional groups (Dai et al., 2025).

In the synchronous spectrum within the range of 500–1700 cm^−1^ (Fig. 3C), two distinct auto-peaks were identified at 1055 cm^−1^ and 1590 cm^−1^. Based on the one-dimensional spectral analysis, these peaks correspond to the C–O–C stretching vibration (1055 cm^−1^) and the asymmetric stretching vibration of C=O in carboxylate groups (1590 cm^−1^), respectively, both of which are characteristic signals introduced by CNF into the LF-CNF complexes. Generally, a redder spectral color indicates higher sensitivity of the corresponding functional group and greater auto-peak intensity. Accordingly, it can be inferred that, under the external perturbation of complexes concentration, the functional group corresponding to the C–O–C stretching vibration at 1055 cm^−1^ is the most sensitive. The cross-peak at 1055 cm^−1^ and 1590 cm^−1^ in the synchronous spectrum is positive, indicating that the variations of these two vibrational modes occur in the same direction. Further analysis of the asynchronous spectrum in this region (Fig. 3D), based on Noda's rules (Noda, 2010) for cross-peak interpretation, reveals that the sequential order of spectral changes is 1055 cm^−1^ > 1590 cm^−1^. This suggests that, with increasing complexes concentration, the C–O–C stretching vibration changes first, followed by the asymmetric stretching vibration of C=O in the carboxylate group.

### 3.3 Interfacial activities

The interfacial properties of LF and CNF at the oil/water interface can be partially characterized by measuring their interfacial tension (Wu et al., 2025b). As shown in Fig. 4A, with an increase in concentration from 0.2 wt% to 1.0 wt%, the interfacial tension of CNF gradually reduced from 26.84 mN/m to 19.84 mN/m; while that of LF decreased from 21.37 mN/m to 13.45 mN/m. Research has shown that the surface tension of protein solutions is concentration-dependent, which is typically related to the adsorption behavior at the interface (Phuc Nghia et al., 2013). Across the entire concentration range, the interfacial tension of LF remained lower than that of CNF; therefore, we infer that the interfacial behavior in the LF-CNF complexes system is likely dominated by LF.

**Fig. 4:**
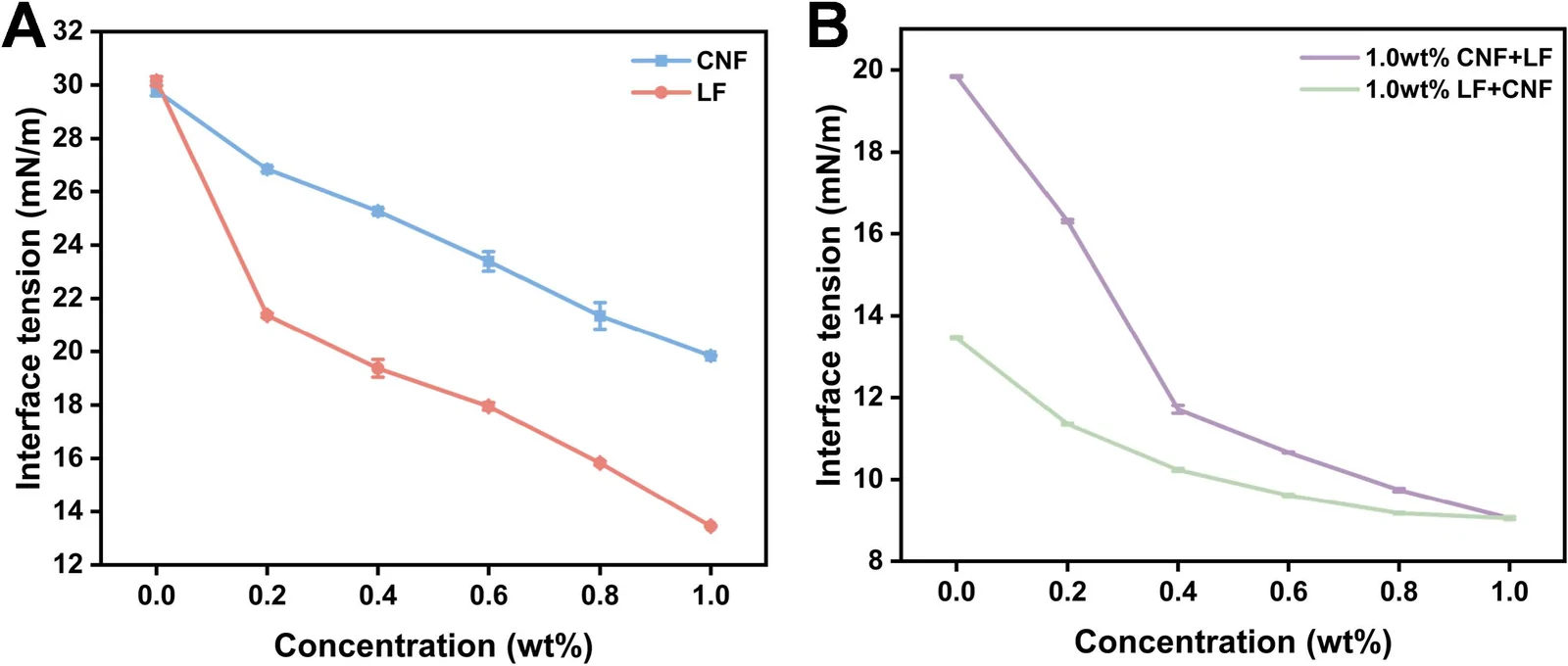
(A) Interfacial tension of individual LF and CNF; (B) Competitive interfacial tension of LF-CNF complex system.

To verify the interfacial behavior of the LF-CNF complexes system, two sets of experiments were conducted: (1) the concentration of CNF was maintained at 1.0 wt%, while LF was incrementally introduced up to a concentration of 1.0 wt%, then measured the interfacial tension; (2) the LF concentration was fixed at 1.0 wt%, while CNF was progressively added up to 1.0 wt%, then measured the correspondin interfacial tensiong. The results are shown in Fig. 4B. Upon introducing LF into the CNF system, the interfacial tension of the complexes exhibited a significant reduction from 19.84 mN/m to 9.05 mN/m. Conversely, the introduction of CNF into the LF system resulted in a reduction of the interfacial tension of the complexes, dropping from 13.45 mN/m to 9.05 mN/m. This result further indicates that LF plays a dominant role in the surface activity of the LF-CNF complexes system. Furthermore, the LF-CNF complexes display a lower oil-water interfacial tension than either LF or CNF alone, indicating that their interaction produces a system with enhanced interfacial activity.

### 3.4 QCM-D

QCM-D is an ultra-sensitive interfacial analysis technique capable of detecting mass changes as small as the nanogram range in real time, as well as providing structural and viscoelastic information about interfacial layers. In recent times, this technique has been extensively utilized to studies of nanoparticle-surface interactions and the adsorption kinetics of polymers, proteins, and biomacromolecules on various substrates. Therefore, it is especially suitable for clarifying the interaction between polysaccharides and proteins at interfaces (Hu et al., 2022). The sensor chip coated with CNF by spin coating was placed in the QCM-D system, and deionized water was introduced until a steady baseline was established. Subsequently, the LF solution was introduced into the system to investigate the electrostatic interactions between the two components. The results are shown in Fig. 5A. After the LF solution was introduced, the apparent adsorbed mass rose rapidly, eventually reaching 1198.05 ng/cm^2^. Subsequent rinsing with deionized water caused slight desorption, and the Δm value remained at approximately 864.96 ng/cm^2^. We then sequentially injected CNF, deionized water, LF, and deionized water to further investigate the adsorption behavior between LF and CNF. Over time, Δm continued to increase, suggesting that LF and CNF have undergone layer-by-layer adsorption via electrostatic interactions, with interaction strengths sufficient to render the process effectively irreversible.

**Fig. 5:**
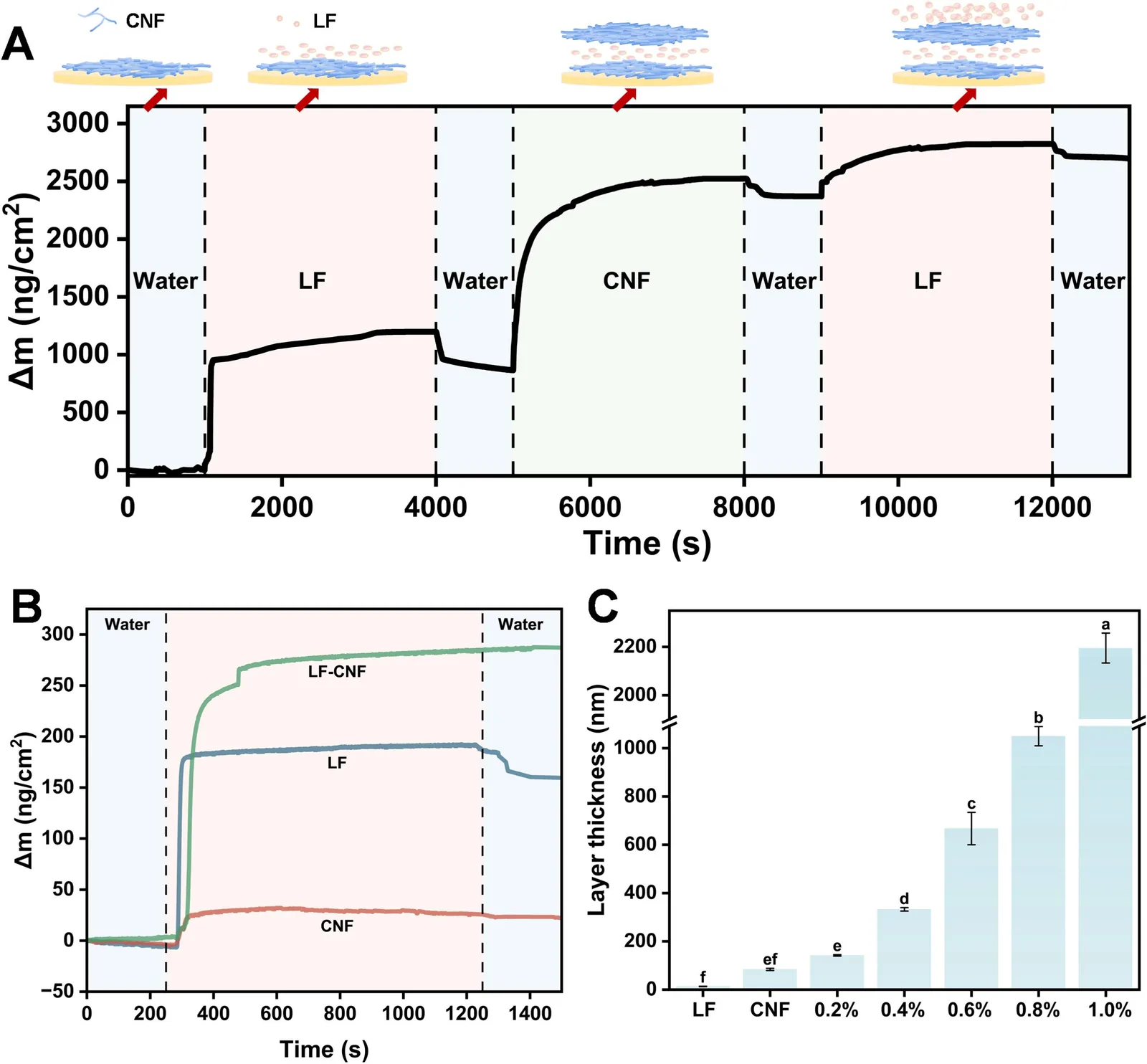
(A) Schematic diagram and adsorption behaviors between LF and CNF; (B) Adsorbed mass (Δm) as a function of time for LF, CNF and LF-CNF complexes at the corn oil-coated surface; (C) Interfacial thickness of LF, CNF, and LF-CNF complexes.

### 3.5 Adsorption behavior

QCM-D was employed to quantify the adsorption mass of LF, CNF, and LF-CNF complexes on a model oil-water interface, thereby providing comparative information on their interfacial adsorption behaviors. In this experiment, a homogeneous oil layer was initially deposited onto the sensor chip surface by spin coating to construct a simplified model oil-water interface. The chip was then placed in the QCM-D system and deionized water was introduced until the signal stabilized. Subsequently, solutions of LF, CNF, and LF-CNF complexes were injected sequentially, and the apparent adsorbed mass (Δm) was calculated by monitoring frequency changes in real time. As shown in Figs. 5B, the time-dependent curves of the adsorption mass for LF, CNF, and the LF-CNF complexes indicate that all three can effectively adsorb onto the oil film surface. Their adsorption kinetics exhibit similar behavior, all show rapid initial adsorption, succeeded by a gradual attainment of equilibrium. This is primarily because the initially introduced particles can adsorb directly onto the exposed oil film surface, whereas subsequent particles must adsorb onto the already formed interfacial layer, where steric hindrance and electrostatic repulsion slow down further adsorption. Subsequently, during the deionized water rinsing process, the adsorption mass initially decreases swiftly, indicating that unbound and loosely bound particles desorb quickly, followed by a slower decline and eventual stabilization, suggesting that loosely bound and unbound particles are desorbing rapidly. It is commonly accepted that a greater mass of adsorption suggests more intense particle adsorption at the interface between oil and water (Yang et al., 2024). Specifically, CNF exhibits weak interfacial activity toward the oil film and is easily partially removed during rinsing, its adsorption capacity was the lowest, ultimately reaching only 22.53 ng/cm^2^. In contrast, owing to its superior emulsifying properties, LF exhibits a stronger adsorption capacity than CNF, with a final apparent adsorbed mass of 159.40 ng/cm^2^. The adsorption capacity of the LF-CNF complexes is significantly higher than that of either component alone, and no apparent desorption is observed during the rinsing process. The final apparent adsorbed amount reaches 287.04 ng/cm^2^, which is approximately 1.8 times and 12 times greater than that of LF and CNF alone, respectively. In summary, the findings suggested that LF-CNF complexes exhibited enhanced adsorption capacity and formed more stable interfacial layers on the model oil-water interface, which may contribute to their improved stabilization performance in O/W Pickering emulsions.

### 3.6 Interfacial thickness

To corroborate the preceding results and enable a quantitative assessment of the interfacial adsorption layer, a model system based on monodisperse polystyrene latex particles was utilized. The apparent interfacial layer thickness was calculated by comparing the size of the latex beads before and after biopolymer adsorption. The hydrophobic surface properties of monodisperse polystyrene latex beads provide a simplified model for evaluating biopolymer adsorption at oil-water interfaces, and compared to polydisperse emulsion systems, the change in particle size after biopolymer adsorption is more pronounced, which helps improve the accuracy of the detection (Li et al., 2022). The interfacial layer thicknesses created by LF and CNF alone were 13.26 nm and 84.02 nm, respectively (Fig. 5C). In contrast, all LF-CNF complexes generated thicker interfacial layers than the individual components, and the layer thickness grew steadily as the complex concentration increased, attaining a maximum value of 2195.23 nm at a concentration of 1.0 wt%. This is because LF and CNF combine through electrostatic interactions, forming a more compact complexes interfacial layer. The thicker interfacial layer is likely to boost the ability of emulsion droplets to withstand external environmental pressures, thereby enhancing their stability (Jiang et al., 2022).

### 3.7 LF-CNF stabilized Pickering emulsion

#### 3.7.1 Stability and particle size

To further demonstrate the specific advantage of LF-CNF complexes over their individual components and simple physical mixing, emulsions stabilized by CNF, LF, and a non-precomplexed physical mixture of LF and CNF (LF-CNF-MIX) were prepared as controls. As shown in Fig. S2A, all freshly prepared emulsions exhibited a uniform and milky-white appearance. After 24 h of storage, obvious phase separation was observed in the emulsions stabilized by CNF and LF alone, whereas the LF-CNF-MIX and preformed LF-CNF complex-stabilized emulsions remained relatively homogeneous. However, the droplet sizes of the LF-CNF-MIX stabilized emulsion(1.89 μm) was significantly higher than that of the emulsion stabilized by the preformed LF-CNF complexes (1.20 μm, Fig. S2B). These results indicate that although simple physical mixing of LF and CNF provides a certain degree of emulsion stabilization, precomplexation of LF and CNF is more effective in promoting the formation of smaller droplets and improving emulsion stability. To further investigate the effect of LF-CNF complex concentration on emulsion stability, emulsions were prepared using LF-CNF complexes at different concentrations and subsequently subjected to a series of characterizations. As shown in Fig. 6A, photographs of the emulsion appearance were taken after 0 and 28 days. All emulsions that were prepared newly displayed a uniform and milky white characteristic. After a storage period of 28 days, emulsions stabilized with 0.2–0.8 wt% concentrations of the complexes exhibited varying degrees of phase separation. Furthermore, as the complexes concentration increased, the height of the creamed water layer progressively declined. This behavior is likely due to an insufficient level of complexes, which is unable to effectively prevent separation between the aqueous phase and the emulsified layer (Wu et al., 2025a). Conversely, the appearance of the emulsion stabilized by 1.0 wt% LF-CNF showed no significant changes; it remained milky white and uniform, demonstrating good stability. This is likely because of the formation of a thicker interfacial layer by the high-concentration LF-CNF complexes, which significantly improves long-term stability.

**Fig. 6:**
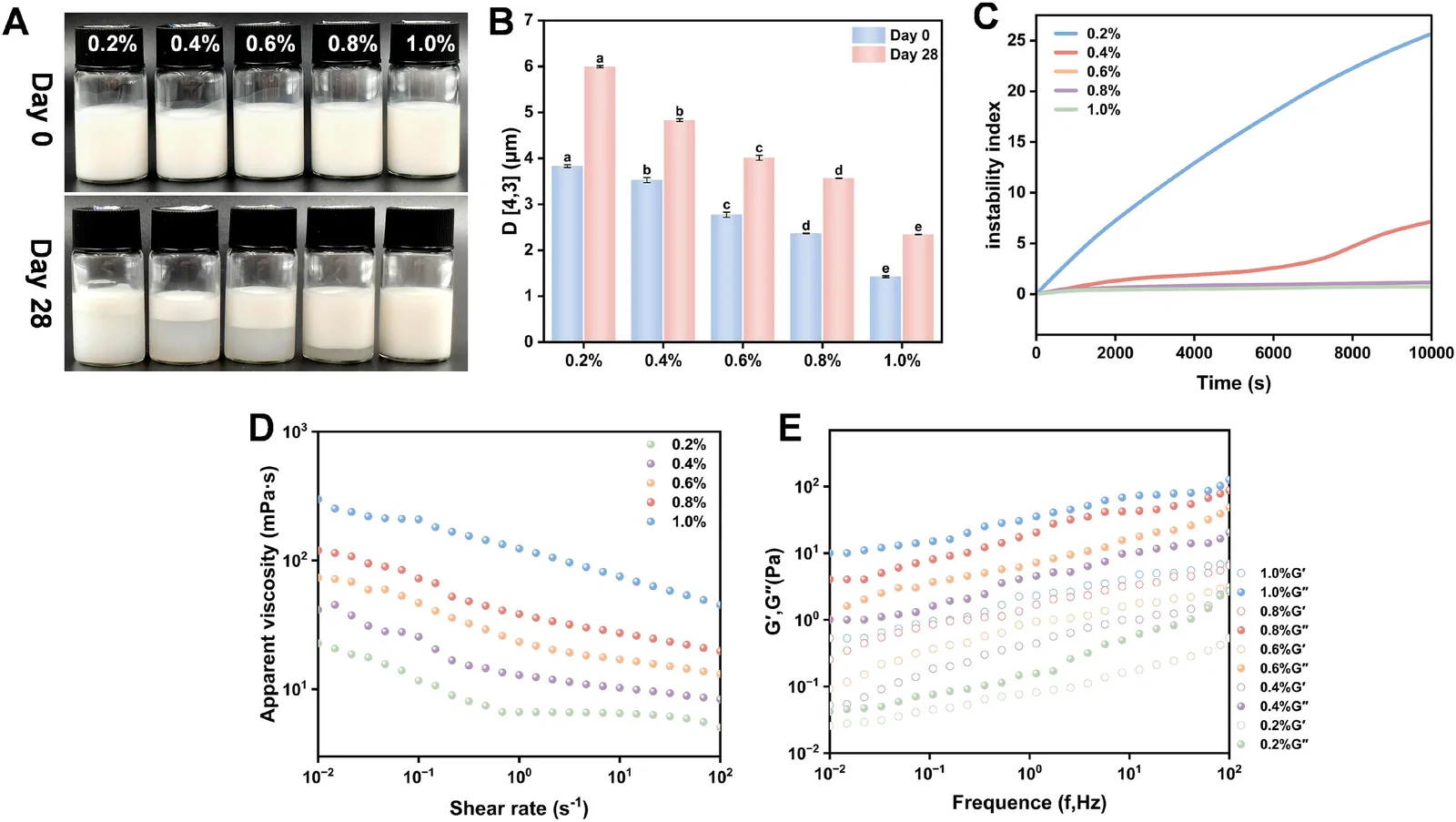
Properties of LF-CNF complexes stabilized Pickering emulsions. (A) Storage stability; (B) Droplet size of the emulsions before and after 28 days and (C) physical stability; (D) Viscosity (E) and moduli of Pickering emulsions containing various LF-CNF complexes concentrations.

The droplet sizes of the emulsion are illustrated in Fig. 6B, comparing day 0 to after 28 days of storage. A pronounced concentration dependence is observed between the emulsion droplet size and complexes concentration. At the initial stage (day 0), with an increase in the concentration of complexes from 0.2 wt% to 1.0 wt%, the droplet size gradually decreases from 3.83 μm to 1.42 μm. After 28 days, the droplet size of each samples increases to some extent, among which the 1.0 wt% group shows the smallest increase (from 1.42 μm to 2.34 μm), whereas the low-concentration samples exhibit more pronounced growth. These results indicate that a higher complexes concentration is more favorable for maintaining emulsion stability, which is consistent with the analysis of emulsion physical stability (Fig. 6C). These confirmed that high-concentration LF-CNF complexes can enhance the stability of emulsions by forming a more stable interfacial structure, thereby yielding Pickering emulsions with superior overall performance.

#### 3.7.2 Rheological behavior

To further investigate the stability and structural characteristics of emulsions, we analyzed their rheological behavior (Zhu et al., 2026). As shown in Fig. 6D, all emulsions exhibited typical shear-thinning behavior, meaning that their apparent viscosity gradually diminished as the shear rate rose from 10^−2^ s^−1^ to 10^2^ s^−1^ (Ma et al., 2022). Meanwhile, the emulsion viscosity exhibited a clear concentration dependence, increasing with the concentration of the complexes. The frequency sweep results are presented in Fig. 6E. Within the frequency range of 10^−2^–10^2^ Hz, the loss modulus (G″) of all emulsions exceeds the storage modulus (G′), indicating that viscous behavior predominates in the emulsion system (Shi et al., 2026). In addition, it can be observed that both the viscosity and modulus of the emulsions increase with increasing complexes concentration. The increase in emulsion viscosity and modulus with increasing complex concentration can be attributed to two possible contributions: (i) adsorption of LF-CNF complexes at the oil–water interface, which strengthens the interfacial film and may promote droplet–droplet interactions; and (ii) the presence of excess non-adsorbed complexes in the continuous phase, which directly raises the viscosity of the aqueous phase. In the present study, the loss modulus (G″) exceeded the storage modulus (G′) across the measured frequency range, indicating that the emulsions exhibited predominantly viscous behavior without a strong elastic network. This suggests that the enhancement of viscosity is mainly governed by the non-adsorbed complexes dispersed in the continuous phase, rather than by a rigid three-dimensional network formed solely through interfacial adsorption. Nevertheless, a weak contribution from adsorbed complexes cannot be entirely excluded.

#### 3.7.3 Microstructure of emulsions

CLSM was employed to visualize the emulsion microstructure, allowing identification of the internal phase composition and the spatial distribution of LF-CNF complexes. Oil phase (red, marked with Nile Red), LF (blue, marked with Nile Blue), and CNF (green, marked with Calcofluor White) were specifically labeled with fluorescent dyes, respectively. The CLSM image (Fig. 7A) shows that red oil droplets are uniformly dispersed in the continuous phase, demonstrating that the emulsion formed is of the oil-in-water (O/W) type. Concurrently, blue (LF) and green (CNF) fluorescence signals were observed on the surfaces of the red oil droplets, indicating that LF and CNF co-adsorbed at the oil-water interface to form a complexes interfacial layer. The presence of green and blue signals within the continuous phase indicated the existence of unadsorbed CNF and LF in the system. From a microstructural perspective, the emulsion stabilized by the 0.2 wt% LF-CNF complexes showed the largest droplet size. In contrast, the emulsion stabilized by the 1.0 wt% LF-CNF complexes had the smallest droplets and the most uniform distribution. This observation is in close agreement with the aforementioned laser particle size analyzer measurements, collectively indicating that the higher complexes concentration facilitates the formation of smaller and more uniform droplets during emulsification, thereby enhancing the stability of the emulsion.

**Fig. 7:**
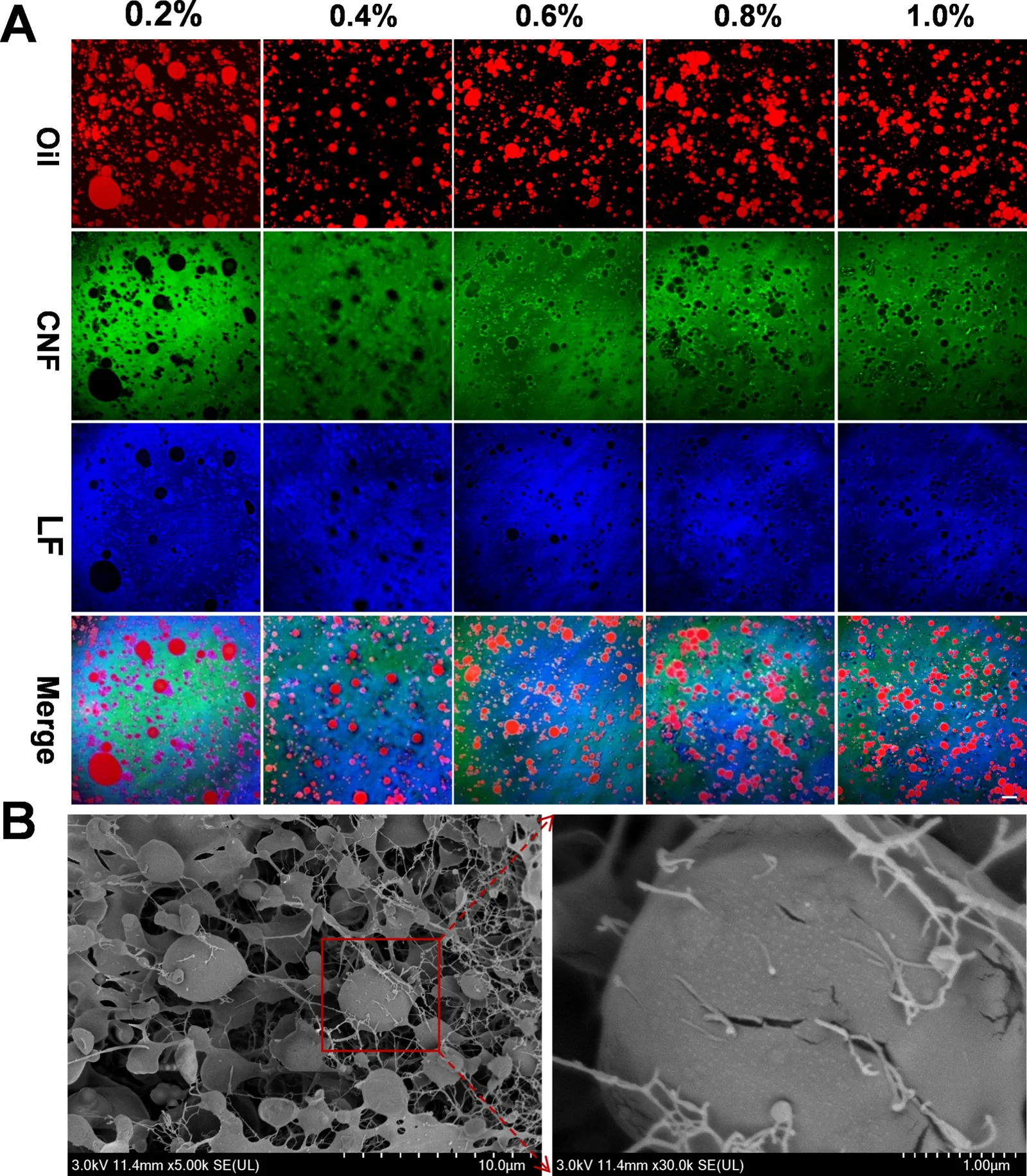
Microstructure of LF-CNF complexes stabilized Pickering emulsions.(A) CLSM micrographs of the emulsions stabilized by LF-CNF complexes with different concentrations (LF: blue channel; CNF: green channel; corn oil: red channel; scale bar: 10 μm); (B) Cryo-SEM images. (For interpretation of the references to color in this figure legend, the reader is referred to the web version of this article.)

Cryo-SEM employs ultra-low-temperature cryofixation technology to preserve liquid samples in a state close to their original condition, thereby enabling direct observation of the microstructure of emulsions (Costa et al., 2021). To investigate the stabilization mechanism in depth, we selected the 1.0 wt% LF-CNF complexes emulsion, which exhibited the best performance, for Cryo-SEM observation. As shown in Fig. 7B, the emulsion exhibits a regular, multilayer parallel layered structure. A substantial number of droplets are evenly distributed across each layer, with the layers connected by an interconnected porous network, resulting in a dense overall structure. This layered interface acts as an effective physical barrier, inhibiting droplet aggregation and coalescence. In high-magnification images, fibrous network connections between droplets can be further observed, revealing a distinct droplet bridging phenomenon, which is associated with the three-dimensional interconnected network created by the interweaving of unadsorbed LF-CNF particles in the continuous phase (Nimaming et al., 2024). Additionally, white dot-like and fibrous substances are observed on the droplet surfaces, which are likely LF and CNF adsorbed onto the droplet surfaces, further corroborating the adsorption characteristics of the complexes at the oil-water interface.

Based on the above microstructural evidence, the possible mechanisms by which LF-CNF complexes stabilize emulsions can be summarized as follows: (1) LF-CNF complexes adsorb at the oil/water interface and form a structured interfacial layer, which may act as a protective barrier to reduce droplet coalescence; (2) Excess LF-CNF complexes remaining in the continuous phase construct a three-dimensional network architecture through fiber-fiber interactions, thereby restricting droplet movement and contributing to the long-term stability of the emulsified system.

#### 3.7.4 pH and ionic stability of emulsions

The impact of ionic strength and environmental pH on the stability of LF-CNF complexes emulsions are crucial for understanding their practical application conditions. First, the effects of pH (2.0, 4.0, 6.0, 8.0, and 10.0) on emulsions stabilized by 1.0 wt% LF-CNF were evaluated. As shown in Fig. 8A, all emulsions that were freshly prepared demonstrated a consistent milky look. After 28 days of storage, severe phase separation was observed only in the sample at pH 2.0, whereas emulsions at other pH values remained homogeneous, indicating good storage stability. The alterations in droplet dimensions (Fig. 8B) aligned with the visual findings, as a notable rise in the droplet size was observed exclusively at pH 2.0, whereas the other groups displayed merely minor fluctuations. This indicates that the emulsions exhibited reduced stability under strongly acidic conditions. This phenomenon may be associated with the altered electrostatic interactions within LF-CNF complexes at low pH. Under strongly acidic conditions, the increased protonation of LF may change the charge balance between LF and CNF, potentially weakening the intermolecular interactions responsible for maintaining the complex structure. As a result, the interfacial adsorption capability and structural stability of LF-CNF complexes may be reduced, leading to droplet aggregation and phase separation. The effect of ionic strength was further investigated by adding NaCl at different concentrations (20–100 mM). Macroscopic images (Fig. 8C), changes in particle size (Fig. 8D) all indicate that, within this range of salt concentrations, ionic strength has no significant effect on emulsion stability. Over the entire 28-day storage duration, none of the examined emulsions showed any noticeable phase separation. This phenomenon suggests that LF-CNF stabilized emulsions exhibit good tolerance to moderate ionic strength conditions within the tested NaCl concentration range. In general, LF-CNF complexes provided good stabilization performance across most tested pH conditions and moderate salt concentrations, suggesting their potential application in food systems. However, further studies under more complex conditions, including higher ionic strength and different food matrices, are still required to fully evaluate their practical applicability.

**Fig. 8:**
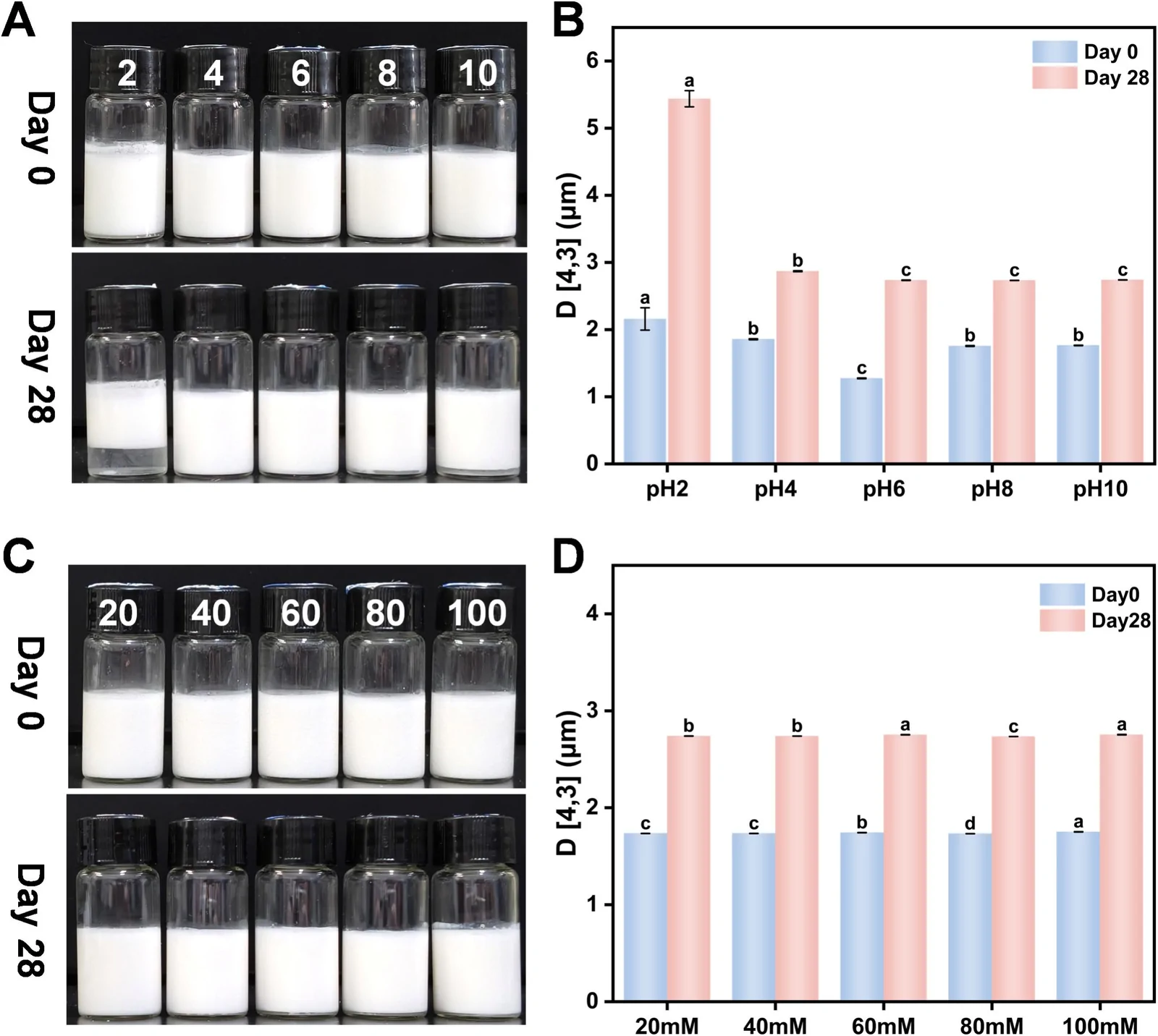
Storage stability and droplet size of the emulsions prepared by LF-CNF complexes at pH 2.0–10.0 (A and B) or under different NaCl concentration (C and D) before and after 28 days.

## 4 Conclusion

In this study, LF-CNF complexes with excellent emulsifying properties were successfully fabricated via electrostatic interactions, and their formation mechanism, interfacial behavior, and structure–function relationships in stabilizing Pickering emulsions were systematically investigated. The results indicated that the complex system exhibited optimal stability at a pH of 6.0 with a mass ratio of LF to CNF of 1:1. FTIR and 2D-COS-FTIR further suggested that, during the interaction between LF and CNF, the most pronounced conformational changes occurred in the amide II band of LF. The QCM-D and interfacial thickness analyses showed that, compared with individual components, the LF-CNF complexes exhibited higher adsorption at the oil–water interface and, with higher concentrations, developed thicker and more compact interfacial layers, implying enhanced mechanical robustness of the interfacial film. When applied to the stabilization of Pickering emulsions, the complexes appeared to facilitate the formation of a sturdy interfacial coating, which contributed to the improved physical stability of the resulting system. At 1.0 wt%, the emulsions displayed a dense and uniform droplet distribution along with favorable viscoelastic properties. Moreover, the prepared emulsions remained stable over a broad pH range (4.0–10.0) and ionic strength conditions (≤100 mM NaCl), demonstrating their potential adaptability under different environmental conditions. Overall, this study provides a comprehensive characterization of LF-CNF complexes as natural Pickering stabilizers, ranging from molecular interactions and interfacial adsorption behavior to macroscopic emulsion performance. The proposed stabilization mechanism is supported by the experimental evidence obtained, although further direct molecular-level investigations, such as molecular dynamics simulations or in situ interfacial spectroscopic techniques, are still required to clarify the precise arrangement and interactions of LF-CNF complexes at the interface. Beyond their role as emulsion stabilizers, LF-CNF complexes provide a promising natural interfacial platform for constructing functional Pickering emulsions. Future studies should further investigate the application of LF-CNF-stabilized Pickering emulsions for encapsulating and delivering sensitive bioactive compounds, including their encapsulation efficiency, gastrointestinal stability, and controlled-release behavior. In addition, structural optimization and evaluation in real food matrices will be necessary to further promote the practical application of LF-CNF-based emulsion systems in functional food formulations.

## CRediT authorship contribution statement

**Runru Xiang:** Writing – original draft, Methodology, Investigation, Formal analysis. **Jiwei Ding:** Methodology. **Xiaohui Wan:** Methodology. **Yufei Yang:** Methodology. **Wentao Zheng:** Methodology. **Mo Li:** Methodology. **Tao Ma:** Writing – review & editing, Methodology, Funding acquisition, Formal analysis, Conceptualization. **Yongfeng Liu:** Supervision, Resources, Funding acquisition.

## Funding

This work was financially supported by 10.13039/501100001809National Natural Science Foundation of China (32402164), Shaanxi science and technology plan projects of China (2024NC-GJHX-30, 2024NC-ZDCYL-03-07), Xi'an city science and technology plan projects of China (24NYGG0018), Xianyang city science and technology plan projects of China (L2024-CXNL-KJRCTD-DWJS-0010), Shaanxi Kangyuan goat dairy products innovation center Co.Ltd. (SXKY25–022) and the Fundamental research funds for the central universities in China (GK202506038).

## Declaration of competing interest

The authors declare that they have no known competing financial interests or personal relationships that could have appeared to influence the work reported in this paper.

## Data Availability

Data will be made available on request.
