# Supplementary material for: Elucidating interfacial-function relationships of lactoferrin-cellulose nanofibril complexes via multidimensional spectroscopy and QCM-D: interfacial anchoring and bulk stabilization mechanisms in robust Pickering emulsions
Source: Food Chem X. 2026 Sep 19;39:104455. doi: 10.1016/j.fochx.2026.104455 (PMC13628691; doi:10.1016/j.fochx.2026.104455)
Supplement: Supplementary file 1 [file mmc1.docx]

**[Supplementary data](https://www.sciencedirect.com/science/article/pii/S0268005X24008130#appsec1)**

**Elucidating interfacial-function relationships of lactoferrin-cellulose nanofibril complexes via multidimensional spectroscopy and QCM-D: interfacial anchoring and bulk stabilization mechanisms in robust Pickering emulsions**

Runru Xiang^a^, Jiwei Ding^a^, Xiaohui Wan^a^, Yufei Yang^a^, Wentao Zheng^a^, Mo Li^b^, Tao Ma^a *^, Yongfeng Liu^a *^

^a^ College of Food Engineering and Nutritional Science, Shaanxi Normal University, Xi’an 710119, Shaanxi, China

^b^ College of Food Science and Nutritional Engineering, China Agricultural University, Beijing 100083, China

***Corresponding author:**

- Tao Ma, College of Food Engineering and Nutritional Science, Shaanxi Normal University, Xi’an 710119, Shaanxi, China. E-mail: mataogz@163.com
- Yongfeng Liu, College of Food Engineering and Nutritional Science, Shaanxi Normal University, Xi’an 710119, Shaanxi, China. E-mail: yongfeng200@126.com

**Figure S1** (A-B)TEM image and size distribution of LF and (C-D) CNF.

**Figure S2** (A) Visual appearance and (B) droplet size of the emulsions prepared by CNF, LF, LF-CNF-MIX and LF-CNF complexes,LF–CNF-MIX represents the physical mixture of LF and CNF.
